# Analysis of Influencing Factors of Junior ICU Nurses’ Recognition and Response Abilities to Clinical Deterioration: A Cross‐Sectional Study

**DOI:** 10.1155/ccrp/3230912

**Published:** 2025-12-20

**Authors:** Xueqin Guo, Xianke Wang, Chenzi Xu, Yuhan Wang, Xin Li, Lijuan Xiong, Huan Jin

**Affiliations:** ^1^ Neurosurgical Intensive Care Unit, Union Hospital, Tongji Medical College, Huazhong University of Science and Technology, Wuhan, Hubei 430022, China, hust.edu.cn; ^2^ Plastic Surgery, Union Hospital, Tongji Medical College, Huazhong University of Science and Technology, Wuhan, Hubei 430022, China, hust.edu.cn; ^3^ Department of Nursing, Union Hospital, Tongji Medical College, Huazhong University of Science and Technology, Wuhan, Hubei 430022, China, hust.edu.cn; ^4^ Anesthesiology, Union Hospital, Tongji Medical College, Huazhong University of Science and Technology, Wuhan, Hubei 430022, China, hust.edu.cn

**Keywords:** clinical deterioration, influencing factor, intensive care unit, junior nurse

## Abstract

**Aim:**

To evaluate the current status and explore influencing factors of junior intensive care unit (ICU) nurses’ recognition and response capabilities to clinical deterioration.

**Design:**

This cross‐sectional study followed the STROBE statement.

**Methods:**

From November 2024 to January 2025, 260 junior ICU nurses from five tertiary hospitals in China were recruited. Data were collected using a validated 25‐item questionnaire spanning six dimensions to assess their recognition and response abilities. SPSS was used for statistical analyses, including descriptive statistics, Kruskal–Wallis H/Mann–Whitney *U* tests, and multiple linear regression to examine differences under sociodemographic and occupational factors.

**Results:**

The total score for recognition and response abilities among 250 junior ICU nurses was 99 (95, 103), with an average item score of 3.96 ± 0.83. Dimension scores ranked from lowest to highest: emergency handling, evaluation, teamwork, disease information analysis, disease information acquisition, and clinical decision‐making. Education level, work experience, and participation in disease observation training were identified as significant influencing factors.

**Conclusions:**

Junior ICU nurses in China demonstrate relatively strong overall observation skills but insufficient clinical decision‐making abilities. Nursing managers and educators should integrate these factors into training to enhance young nurses’ capabilities in recognizing and responding to clinical deterioration, which is crucial for improving critical care outcomes.

**Patient or Public Contribution:**

Patients and the public were not directly involved in the design, implementation, or reporting of this study. However, the results of the study emphasize the importance of improving primary ICU nurses’ clinical decision‐making skills and emergency response skills, which may have an impact on patient care.

## 1. Background

Patient safety represents a core concern within global healthcare systems, and the timely identification and management of clinical deterioration are pivotal measures to ensure patient safety. Nevertheless, nursing continues to face persistent challenges in patient safety monitoring, particularly concerning delays in recognizing and responding to clinical deterioration [1]. Clinical deterioration is characterized as the transition from a stable condition to a critical pathological state [2]. The undetected progression of such deterioration significantly increases mortality rates, the incidence of adverse events, and healthcare costs. Globally, over half (51.2%) of adverse events are considered preventable [3], with 11% of patient deaths attributed to clinical inaction or insufficient awareness of deterioration [4].

Intensive care unit (ICU) patients benefit significantly from continuous physiological parameter monitoring and advanced medical interventions [5], which contribute to a reduction in patient mortality compared with general wards [6]. Currently, early warning scoring systems and standardized tools (e.g., NEWS [7] and SOFA scores [8]) offer objective clinical assessments through systematic vital sign monitoring. However, their limited sensitivity to atypical symptoms and inherent monitoring delays necessitate further improvement [9]. Notably, as frontline clinical observers, nurses’ professional intuition and clinical experience play irreplaceable roles in detecting subtle pathological changes [10, 11]. Assessment tools based on nursing intuition, such as the Nurse Intuition Patient Deterioration Scale [12] and the Attitudes Toward Recognizing Early and Noticeable Deterioration Scale [13], partially address the limitations of objective metrics. Nevertheless, their effectiveness is highly contingent upon the nurses’ analytical capabilities and clinical judgment.

Nurses require effective observational skills and reasoning capabilities to formulate rational and reliable clinical judgments throughout the processes of assessment, diagnosis, implementation, and evaluation [14]. However, challenges persist in nurses’ recording of vital signs and their application of these signs to activate rapid response systems [15]. Factors such as cognitive biases [16] and perceptual barriers [17] can result in insufficient or delayed activation of rapid response teams, thereby negatively impacting both patient outcomes and organizational performance [18]. Junior nurses face unique challenges in this domain. As practitioners transitioning from nursing students to professional roles, they often struggle to effectively manage clinical deterioration events due to limited experience, inadequate knowledge translation, and the influence of high‐pressure working environments [19]. A Malawian study [20] involving 322 junior nurses reported median competency scores of 71 points (IQR: 68–75) in deterioration recognition and management. These scores were significantly influenced by work experience, departmental characteristics, and exposure to training.

With the rapid increase in global demand for patient safety and the aging population, the burden of critical illness has become more significant than commonly recognized [21]. The number of critically ill patients admitted to ICUs continues to rise. Understanding and addressing the factors that influence junior ICU nurses’ ability to identify and respond to deteriorating conditions will be crucial to strengthening patient safety practices and ensuring high‐quality care for patients. To date, research on the clinical deterioration identification and response capabilities of junior nurses in Chinese ICUs remains unexplored. Clarifying the current state of this group’s competencies and their influencing factors holds substantial practical value for optimizing nursing training systems and enhancing patient safety defenses. This cross‐sectional study aims to delineate the competency levels of Chinese junior ICU nurses in recognizing and managing clinical deterioration, identify associated influencing factors, and provide evidence‐based support for developing targeted strategies to enhance these competencies.

## 2. Methods

### 2.1. Study Design, Setting, and Participants

A cross‐sectional descriptive study utilizing convenience sampling was conducted from November 2024 to January 2025. The study focused on ICU nurses from five tertiary hospitals located in Wuhan, Shanghai, Shandong, Fujian, and Chongqing, China. As of 2024, these hospitals had inpatient bed capacities ranging from 3000 to 6400. The inclusion criteria were as follows: nurses currently working in the ICU with ≤ 5 years of ICU experience, those independently and directly involved in patient care, and those who voluntarily agreed to participate in the study. Nurses on internships and those on long‐term leave (≥ 3 months) were excluded. The sample size was estimated using the Kendall method, which recommends a sample size of 5–10 times the number of scale items. Considering a potential invalid questionnaire rate of 20%, the final sample size was determined to be between 156 and 313 participants.

### 2.2. Measures

#### 2.2.1. Demographic Information Questionnaire

This questionnaire is designed to collect personal and work‐related information from the surveyed nurses. Personal information includes gender, age, marital status, education level, and motivation for major selection. Work‐related information encompasses department, years of experience, technical title, monthly income, employment mode, number of night shifts per month, average nurse–patient ratio, and whether they have participated in training related to patient condition observation. Work‐related information incorporates factors identified in previous studies that may influence nurses’ work engagement and professional attitudes.

#### 2.2.2. Junior Nurses’ Recognition and Response Abilities to Clinical Deterioration (RRCD) Scale

This scale was developed by Laiyu Xu et al. [19] in the context of Chinese nursing practice. Additionally, we have obtained the Chinese version of the scale from the corresponding author of the original study. It was created and validated through a literature review, qualitative interviews, and Delphi expert consultations. The scale is primarily used to assess the RRCD abilities of novice nurses. Permission to use this tool was obtained from the developers, and the Chinese version of the questionnaire was acquired for this study. The scale consists of 25 items divided into six dimensions: emergency‐handling ability (four items), clinical decision‐making ability (five items), teamwork ability (4 items), disease information acquisition ability (seven items), disease information analysis ability (three items), and evaluation ability (two items). A 5‐point Likert scale is used, with scores ranging from 1 to 5 representing “*strongly disagree*,” “*disagree*,” “*not sure*,” “*agree*,” and “*strongly agree*,” respectively. Items 6, 12, and 20 are reverse‐scored, and their scores need to be converted (e.g., in a 5‐point scale, 1 is converted to 5, 2 to 4, etc.) to align with the direction of positive scoring. These adjusted scores are then added to the scores of the other items to calculate the total score. The total score ranges from 25 to 125. Previous studies have shown that the scale has good reliability and validity. The six factors explained 69.310% of the total variance, with a Cronbach’s *α* coefficient of 0.905 and a content validity index of 0.878. In this study, the Cronbach’s *α* coefficient was 0.757, indicating acceptable internal consistency.

### 2.3. Data Collection

The questionnaire was created using an online survey platform (http://www.wjx.cn/). Researchers established contact with the participating hospitals and obtained approval from the nursing departments to conduct the study. Subsequently, the online questionnaire links were distributed directly to the nurses.

To ensure informed consent, all participants were fully briefed before the study began, and the questionnaire was set to start only after their agreement was obtained. Written informed consent was obtained from all participants. The study adhered to the guidelines of the Declaration of Helsinki. Participants were instructed to complete the questionnaires voluntarily and independently. They were also assured of the anonymity of their responses and their right to withdraw from the study at any time without facing any consequences. A standardized guide was provided to explain the study’s objectives and the method for answering the questions. To maintain data integrity, each ID account was permitted to respond only once, and only fully completed questionnaires could be submitted.

A total of 260 questionnaires were collected. The content of the questionnaires was verified by two independent researchers. Since the pretest indicated that a minimum of 2 min was required to complete the questionnaire, five responses with completion times of less than 2 min were excluded. Additionally, five questionnaires with obvious response patterns or logical inconsistencies were removed. Ultimately, 250 valid questionnaires were retained, yielding a valid response rate of 96.15%.

### 2.4. Data Analysis

The data were directly exported from the questionnaire platform and analyzed using SPSS 24.0 (IBM Corporation). After data entry, rigorous verification and cleaning procedures were conducted to ensure data quality. No missing data were identified during this process. The Kolmogorov–Smirnov test revealed that all continuous variables exhibited nonnormal distributions. Descriptive statistical methods were applied to characterize the sample: categorical variables were summarized as frequencies and percentages, while nurses’ scores for recognizing and responding to clinical deterioration were expressed as medians and interquartile ranges (*P*_25_–*P*_75_). To assess differences between levels of various factors, nonparametric tests were utilized: the Kruskal–Wallis *H* test for multigroup comparisons and the Mann–Whitney *U* test for two‐group comparisons. The assumptions of the multiple linear regression model—linearity, independence, homoscedasticity, and normality of residuals—were tested and satisfied. Based on variables showing statistical significance in univariate analysis, stepwise multiple linear regression (with *α* in = 0.05 and *α* out = 0.10) was performed to identify key factors influencing junior ICU nurses’ ability to recognize and respond to clinical deterioration. The total score for recognizing and responding to clinical deterioration was designated as the dependent variable, while significant variables from the univariate analysis were included as independent variables in the regression model. All statistical tests were two‐tailed, with the significance level set at 0.05.

### 2.5. Ethical Considerations

The study was approved by the Ethics Committee of Union Hospital, Tongji Medical College, Huazhong University of Science and Technology (no. 0466) and ensured the anonymity and confidentiality of the respondents.

## 3. Results

### 3.1. Relationship Between Nurse Characteristics and RRCD

There were 360 junior nurses working in the ICUs of the five hospitals. Due to various reasons such as work schedules and leave, a total of 260 nurses participated in the survey, resulting in an external dropout rate of 27.78%. No significant differences were observed in general demographic data between nurses who completed the questionnaire and those who did not (*p* > 0.05), indicating a low risk of selection bias. A total of 250 junior ICU nurses were ultimately enrolled in the study. The majority of participants were female (66.80%), unmarried (95.60%), and held a bachelor’s degree (89.60%). The participants’ ages ranged from 22 to 30 years, with an average age of 25.28 years (standard deviation [SD]: 2.04). Their work experience varied between 1 and 5 years, with an average of 2.65 years (SD: 1.53). Additionally, 86.00% of the participants had received training in disease observation. The results demonstrated that several factors significantly influenced nurses’ ability to identify clinical deterioration (*p* < 0.05, Table 1). These factors included age, education level, work experience, employment mode, technical title, frequency of night shifts, monthly income, and participation in disease observation training.

**Table 1:** Relationship between nurse characteristics and RRCD (*n* = 250).

Characteristics	*N* (%)	*M* (P_25_, P_75_)	*z/H*	*P*
Gender^a^				
Male	83 (33.20)	99.00 (95.50, 103.00)	−0.315	0.753
Female	167 (66.80)	99.00 (94.50, 103.50)		
Age^b^				
22–24	105 (42.00)	96.00 (92.00, 99.50)	62.316^∗∗^	**< 0.001**
25–27	102 (40.80)	100.00 (96.75, 104.00)		
28–30	43 (17.20)	106.00 (100.00, 110.00)		
Marital status^b^				
Married	11 (4.40)	99.00 (96.00, 105.00)	−0.329	0.742
Unmarried	239 (95.60)	99.00 (95.00, 103.00)		
Education level^b^				
College degree or below	9 (3.60)	92.00 (81.00, 97.50)	9.001^∗^	**0.011**
Bachelor’s degree	224 (89.60)	99.00 (95.25, 104.00)		
Master’s degree or above	17 (6.80)	98.00 (93.00, 106.50)		
Years of experience^b^				
1	89 (35.60)	95.00 (91.00, 98.50)	106.302^∗∗^	**< 0.001**
2	45 (18.00)	99.00 (96.00, 103.50)		
3	33 (13.20)	98.00 (96.00, 101.00)		
4	37 (14.80)	103.00 (96.50, 106.50)		
5	46 (18.40)	107.00 (101.75, 111.25)		
Department^b^				
Emergency lCU	40 (16.00)	98.00 (94.50, 103.50)	6.999	0.136
Internal medicine lCU	73 (29.20)	100.00 (98.00, 103.75)		
Surgery lCU	69 (27.60)	99.00 (96.00, 103.00)		
Pediatric lCU	27 (10.80)	97.00 (93.00, 104.00)		
General lCU	41 (16.40)	99.00 (94.00, 107.00)		
Employment mode^b^				
Labor dispatch	46 (18.40)	99.00 (92.75, 105.00)	7.190^∗^	**0.027**
Contract system	185 (74.00)	98.00 (95.00, 102.50)		
Formal establishment	19 (7.60)	103.00 (97.00, 110.00)		
Technical title^b^				
Junior nurse	114 (45.60)	96.00 (92.75, 99.00)	67.918^∗∗^	**< 0.001**
Junior nurse practitioner	119 (47.60)	101.00 (97.00, 105.00)		
Supervising nurse practitioner	17 (6.80)	107.00 (100.50, 111.00)		
Night shift frequency(month)^b^				
0	10 (4.00)	95.00 (89.25, 96.50)	14.096^∗^	**0.003**
1–4	165 (66.00)	100.00 (95.50, 105.00)		
5–8	74 (29.60)	98.00 (94.00, 101.50)		
> 8	1 (0.40)	—		
Monthly income^b^				
≤ 5000	27 (10.80)	98.00 (93.00, 99.00)	13.746^∗^	**0.003**
5001–10000	149 (59.60)	98.00 (94.00, 103.50)		
10,001–15000	69 (27.60)	100.00 (97.00, 105.00)		
> 15,000	5 (2.00)	108.00 (97.50, 110.50)		
Motivation for major selection^b^				
Personal interest	36 (14.40)	100.00 (96.00, 103.00)	2.986	0.394
Teacher recommendation	50 (20.00)	101.00 (94.25, 106.00)		
Parental recommendation	99 (39.60)	98.00 (94.00, 104.00)		
College admission adjustment	65 (26.00)	98.00 (95.00, 101.00)		
Nurse–patient ratio^b^				
1:2	3 (1.20)	97.00 (89.50, 102.50)	7.638	0.054
1:3	117 (46.80)	100.00 (95.50, 105.00)		
1:4	117 (46.80)	98.00 (95.00, 102.00)		
1:4+	13 (5.20)	96.00 (92.00, 99.00)		
Training in disease observation^a^				
Yes	215 (86.00)	100.00 (96.00, 105.00)	−5.213^∗∗^	**< 0.001**
No	35 (14.00)	95.00 (91.00, 98.00)		

*Note:* *z*: standardized Mann–Whitney U value; H: Kruskal–Wallis test statistic. The bold *p* values are those < 0.05, highlighted to quickly identify variables whose differences are statistically significant.

^a^Mann–Whitney *U* test.

^b^Kruskal–Wallis H test.

^∗^ *p* < 0.01.

^∗∗^ *p* < 0.001.

### 3.2. Scores of RRCD Among Junior ICU Nurses

The total score (the sum of the scores of all items) of RRCD among junior ICU nurses was 99 (95, 103) points, with an average item score of 3.96 ± 0.83. Among all dimensions, emergency‐handling ability demonstrated the highest score (*M* = 4.32 and SD = 0.76), while clinical decision‐making ability showed the lowest score (*M* = 3.76 and SD = 0.88) (Table 2).

**Table 2:** Scores of RRCD among junior ICU nurses (*n* = 250).

Dimensions	Total score *M* (p25, p75)	Average item score ($\bar{\text{x}}$ ± SD)
Emergency‐handling ability	16 (18, 19)	4.32 ± 0.76
Clinical decision‐making ability	19 (18, 20)	3.76 ± 0.88
Teamwork ability	16 (15, 16)	3.93 ± 0.73
Disease information acquisition ability	27 (25, 29)	3.87 ± 0.90
Disease information analysis ability	12 (11, 12)	3.91 ± 0.70
Evaluation ability	8 (8, 9.5)	4.22 ± 0.70

### 3.3. Multiple Linear Stepwise Regression Analysis on Factors Influencing RRCD

The stepwise regression model analysis indicates that educational level (Bachelor’s: *β* = 0.201, *p* = 0.009; Master’s: *β* = 0.273, *p* < 0.001), years of clinical work experience (*β* = 0.603, *p* < 0.001), and experience with disease observation training (*β* = 0.234, *p* < 0.001) together serve as key predictive factors for the ability to recognize and respond to clinical deterioration. The model is statistically significant (*F* = 56.581, *p* < 0.001), and its coefficient of determination (*R* ^2^ = 0.480) along with the adjusted coefficient of determination (adj.*R* ^2^ = 0.472) suggest that these variables collectively account for 48.0% of the variance in the outcome variable (Table 3).

**Table 3:** Multiple linear stepwise regression analysis on factors influencing RRCD (*n* = 250).

Variables	*B* (95% CI)	SE	*β*	*t*	*P*
Constant	82.656 (78.880, 86.432)	1.917		43.116	< 0.001
Education: *R* = associate degree					
Bachelor’s degree	4.743 (1.172,‐8.314)	1.813	0.201	2.616	0.009
Master’s degree	7.804 (3.535,12.073)	2.167	0.273	3.601	< 0.001
Years of experience	2.837 (2.397,3.276)	0.223	0.603	12.713	< 0.001
Training in disease observation: *R* = No					
Yes	4.865 (2.965, 6.765)	0.965	0.234	5.043	< 0.001

*Note:* B: unstandardized coefficients; *β*: unstandardized regression coefficient.

Abbreviations: CI, confidence interval; SE, standard error.

## 4. Discussion

This study investigated self‐reported RRCD among junior ICU nurses. The analysis revealed a median competence score of 99 (95–103) on standardized assessment measures. Using a score of 70 as the threshold to distinguish between good and poor ability [20], the junior ICU nurses in this study generally demonstrate a high level of ability to identify and respond to clinical deterioration. This finding is higher than that reported in a recent study of nurses in tertiary hospitals in Malawi [20]. This may be attributed to the fact that the participants in this study are ICU nurses, who are the first to recognize changes in a patient’s condition [22]. They operate in a more demanding environment compared with general nurses, requiring extensive specialized critical care training and experience in managing emergencies [23]. The differences observed between the two studies may also stem from cultural and contextual factors. In China, the medical environment has better resources, with more advanced training methods and medical care systems, providing nurses with good opportunities for training and development [24].

The emergency‐handling ability of junior ICU nurses scored the highest; this may be attributed to the professional training that nurses receive. Experience in managing emergency situations is widely regarded as a critical foundation for acquiring the skills necessary to provide appropriate care [25]. Particularly for junior ICU nurses, there is an urgent need for training in preparing to use defibrillators, administering cardiopulmonary resuscitation (CPR)‐related medications, and other emergency response competencies [26]. Therefore, during the training process, it is essential to include instruction in cardiac monitoring, arrhythmia diagnosis, CPR, and related areas [27]. Such training enhances the emergency‐handling capabilities of junior ICU nurses, enabling them to respond more confidently and effectively in emergency situations.

Although decision‐making has been identified as a component of professional competence [28], the decision‐making ability of junior ICU nurses scored the lowest, which is consistent with the study by Heba Mohamed Al Anwer Ashour et al. [29]. This may be because experienced nurses have a good adaptation to most clinical practices and can better integrate professional knowledge with practical experience. In contrast, novice nurses have a higher reliance on intuition in clinical decision‐making, which is less accurate [30]. When facing complex clinical decisions, they may be more inclined to avoid or depend on others rather than actively and rationally analyze and make decisions [31]. Therefore, more training and experience accumulation are needed.

Among all items, Item 22 “I can skillfully operate specialized instruments and equipment, such as blood glucose meters, sphygmomanometers, resuscitation bags, ECG monitors, defibrillators, ventilators, and microinfusion pumps” received the highest score of 4.53 ± 0.62. This indicates that junior ICU nurses demonstrate remarkable proficiency in operating specialized medical equipment. This finding is consistent with the research by Shaoman Lin et al. [32]. Nurses receive training in standardized equipment management, enabling them to better master the use of medical devices, enhance their operational proficiency and accuracy, and thereby improve the quality of nursing care.

Item 6 “I cannot perform a correct physical examination of a patient and identify positive signs” (reverse‐scored) had the lowest score of 2.76 ± 0.95. This may reflect the deficiency of junior ICU nurses in physical examination and sign identification. There is now growing evidence that inadequate physical assessment practices by nurses have failed to identify patients at risk of clinical deterioration [33]. Nurses do not practice physical assessment or practice only a little. Over‐reliance on others and technology [34], lack of awareness of the importance of physical examination [35], and difficulty in learning [36] are barriers to physical assessment practice. Research indicates that when nurses focus on systematic physical examination at the beginning of their shift, it can effectively reduce the delay in identifying patient deterioration [37]. Therefore, it is imperative to take effective measures to enhance the physical examination skills of junior ICU nurses.

The level of education is one of the factors influencing RRCD among junior ICU nurses. The higher the level of education, the better the ability to RRCD. A plausible explanation was given in a systematic review that higher levels of education and clinical training gave nurses a more comprehensive and deeper understanding of nursing theory, practice, and the health care system. Higher education broadens nurses’ perspectives on patient care, enabling them to assess complex healthcare situations, identify critical issues, and develop comprehensive, evidence‐based care plans [38]. Administrators should incentivize nurses to continue to improve their education and increase their motivation to participate in continuing education.

Work experience is a critical predictor of the ability of junior ICU nurses to recognize and respond to clinical deterioration in patients. Junior ICU nurses with more extensive work experience demonstrate significantly stronger capabilities compared with their less experienced counterparts, which is consistent with the findings reported by Heather DeGrande et al. [28]. Intuition plays an essential role in detecting subtle and difficult‐to‐quantify changes in a patient’s condition, while clinical experience serves as the cornerstone for developing such intuition and professional expertise. With substantial clinical experience, nurses are able to rapidly comprehend, evaluate, and address complex clinical situations through intuitive or subconscious decision‐making mechanisms [39]. Therefore, educators and administrators should consider designing transitional practice programs for nurses new to the intensive care. These programs may include sessions where experienced nurses share their insights, as well as structured exposure to common emergency clinical scenarios encountered in critical care, thereby accelerating the professional development of novice nurses.

Participation in clinical RRCD training is one of the key factors in improving the competencies associated with junior ICU nurses, and nurses who have received such training are significantly better at RRCD than those who have not been trained. Due to insufficient training in RRCD, junior ICU nurses may fail to acquire critical skills and knowledge, which affects their ability to respond quickly to changes in condition and, consequently, treatment outcomes. Effective educational strategies can enhance nurses’ practice in recognizing and managing clinical deterioration [40]. In South Korea, 47.1% and 41.2% of new ICU nurses reported inadequate vocational training opportunities and content [26]. In our study, 86% of the nurses received relevant training, compared with the majority of primary nurses in Malawi who reported never having received RRCD training [20]. Hospitals need to focus on and provide organizational support to ensure that nurses have access to up‐to‐date training in clinical deterioration recognition and management. These trainings should be flexible, well‐planned, and supported by the work environment to reduce barriers to nurse participation [23, 41]. Training methods may include innovative approaches such as escape room teaching method [42], simulation education programs [43], and virtual patient simulation [44], while training content should be based on evidence‐based medicine and best practices [45] to develop nurses’ clinical reasoning skills.

Having discussed the individual factors of nurses, we must recognize that organizational culture and policy support are equally critical dimensions profoundly influencing the RRCD ability of junior ICU nurses. Hospital‐level factors, such as human resource allocation [46, 47], team communication [48], and a culture that encourages reporting and learning from adverse events rather than solely attributing blame [49], directly impact nurses’ workload, psychological state, and opportunities for learning and reflection from practice. Simultaneously, policy direction is equally crucial. For instance, whether there is support for the operation of long‐term educational programs such as simulation training centers [50] and preceptorship/mentorship programs [51] will determine the sustainability and depth of capacity building at a systemic level. The existing difficulty in transferring learning from training to clinical practice is also essentially a manifestation of insufficient organizational support [52]. Therefore, improvement efforts will be significantly limited if they focus solely on individual nurse development while neglecting the ecosystem in which they operate.

## 5. Limitations

This study has several limitations that may affect the reliability of the results. First, the sample was drawn exclusively from a limited number of hospitals in China, which may restrict the generalizability of the findings to other healthcare settings or regions. Future research could expand the sample size to include nurses from a wider range of hospitals across different regions and types, enabling a more comprehensive exploration of influencing factors. Second, as this study employed a cross‐sectional design, it can only reveal associations between variables rather than establish causal relationships. Future studies could consider adopting longitudinal research designs to further validate potential causal mechanisms among variables. Additionally, data were collected through online questionnaires, and although we filtered responses based on completion time, there may still be issues such as social desirability bias or subjective interpretation by respondents. Future research could incorporate multiple data collection methods (e.g., interviews or observational approaches) to enhance the accuracy and reliability of the data.

## 6. Conclusions

This study demonstrates that junior ICU nurses could further improve their ability to recognize and respond to changes in patient condition, particularly in clinical decision‐making. Factors such as the education level, years of clinical work experience, and experience with disease observation training significantly influence their ability to identify and respond to clinical deterioration. Therefore, we recommend implementing a structured preceptorship program and a targeted RRCD training initiative within the ICU to strengthen nurses’ professional competence. Concurrently, nurses should be encouraged to pursue continuing education and academic advancement, while their clinical experiences are systematically documented and shared within the team. Educationally, a tiered curriculum emphasizing scenario‐based simulation and case debriefing should be developed; clinically, a formal mentorship model coupled with periodic skills assessment should be introduced. For research, future studies should explore cross‐cultural differences in junior ICU nurses’ cognitive and behavioral responses to patient deterioration, identify existing barriers, and design tailored capacity‐building interventions, thereby elevating the overall professionalism of the ICU nursing workforce.

## Disclosure

The authors reviewed and edited the content as needed and take full responsibility for the content of the publication.

## Conflicts of Interest

The authors declare no conflicts of interest.

## Author Contributions

Xueqin Guo: conceptualization, methodology, and writing–original draft. Xianke Wang: investigation, supervision, and writing–review and editing. Chenzi Xu: data curation, validation, and writing–review and editing. Yuhan Wang: formal analysis, visualization, and writing–review and editing. Xin Li: methodology, software, and writing–review and editing. Lijuan Xiong: project administration, resources, and writing–review and editing. Huan Jin: conceptualization, funding acquisition, and writing–review and editing. Xueqin Guo and Xianke Wang contributed equally and should be considered co‐first authors.

## Funding

This study was supported by the 2024 Intra‐Hospital Scientific Research Fund of Union Hospital, Tongji Medical College, Huazhong University of Science and Technology—Special Fund for Pharmacy, Technology, and Nursing Project (2024XHYN081) and 2025 Hubei Provincial Natural Science Foundation (Grant no. 2025AFB682).

## Data Availability

The data that support the findings of this study are available from the corresponding author upon reasonable request.
